# Complete remission of small cell neuroendocrine carcinoma of the cervix treated with chemotherapy of paclitaxel, carboplatin, bevacizumab and pembrolizumab: A case report

**DOI:** 10.1016/j.gore.2026.102110

**Published:** 2026-05-25

**Authors:** Reisa Kakubari, Yui Nonokawa, Yasuyuki Morita, Masayo Hosokawa, Keisuke Ogimoto, Eri Yamabe, Miho Kitai

**Affiliations:** Department of Gynecologic Oncology, Osaka International Cancer Institute, Osaka, Japan

**Keywords:** Cervical cancer, Small cell neuroendocrine carcinoma, Chemotherapy, Pembrolizumab, Case report

## Abstract

**Background:**

Small cell neuroendocrine carcinoma (SCNEC) of the cervix is a rare and aggressive histological subtype associated with a poor prognosis. Here, we report a case of stage IVB cervical SCNEC that achieved a complete remission following first-line treatment with a combination of chemotherapy, anti-angiogenic therapy, and immune checkpoint inhibition.

**Case presentation:**

A 42-year-old woman presented with genital bleeding and vaginal discharge. Computed tomography (CT) revealed a cervical lesion with peritoneal dissemination and multiple subcutaneous metastases. Positron emission tomography-computed tomography (PET-CT) confirmed *para*-aortic lymph node involvement and widespread metastatic disease. Cervical biopsy confirmed SCNEC, with positive immunohistochemical staining for synaptophysin and CD56.

The patient received first-line systemic therapy consisting of paclitaxel and carboplatin, in combination with bevacizumab and pembrolizumab. A total of seven cycles were administered, with no life-threatening adverse events. Follow-up PET-CT demonstrated resolution of previously identified lesions, consistent with a complete remission. At 15 months following initiation of therapy, the patient remains on maintenance treatment with bevacizumab and pembrolizumab, with no evidence of disease progression to date.

**Conclusion:**

This case highlights the potential efficacy of immune checkpoint inhibitor-containing multimodality treatment regimens in the management of advanced cervical SCNEC.

## Highlights

• Stage IVB cervical SCNEC with complete response to ICI-based combination chemotherapy.
• PD-L1 expression and CPS ≥1 may be associated with response to pembrolizumab.
• Bevacizumab may enhance antitumor immunity in combination therapy.
• ICI-including regimens may be a potential option for advanced cervical SCNEC.

## 1 Introduction

Cervical small cell neuroendocrine carcinoma (SCNEC) is an uncommon and highly aggressive subtype of cervical cancer, characterized by rapid progression, early distant metastasis, and poor clinical outcomes. Because of its rarity, evidence-based treatment strategies still remain limited, and current management is largely extrapolated from small cell lung cancer. Patients with advanced-stage disease, particularly those presenting with distant metastases such as peritoneal dissemination, generally have a poor prognosis.

Recent reports have suggested a potential role for immune checkpoint inhibitors in neuroendocrine carcinomas; however, data specific to cervical SCNEC are extremely scarce.

Here, we describe a patient with FIGO stage IVB cervical SCNEC who presented with FDG-avid cervical and peritoneal lesions and achieved a remarkable and durable response to ICI-combination chemotherapy. This case highlights the potential therapeutic value of immune checkpoint inhibition in advanced cervical SCNEC.

## 2 Case presentation

A 42-year-old woman presented to the Gynecologic Oncology department with a several month history of genital bleeding and increased vaginal discharge. A 3.2 cm macroscopic tumor was identified in the uterine cervix, assessed on the sagittal pelvic MRI sequence. Pelvic examination revealed right-sided parametrial invasion, without direct involvement of the vaginal wall. Whole-body computed tomography (CT) scan and positron emission tomography-computed tomography (PET-CT) revealed peritoneal dissemination and multiple subcutaneous metastasis, including the chest, shoulder or lower back (Fig. 1a, b). Brain metastasis was excluded by brain magnetic resonance imaging (MRI).

**Fig. 1:**
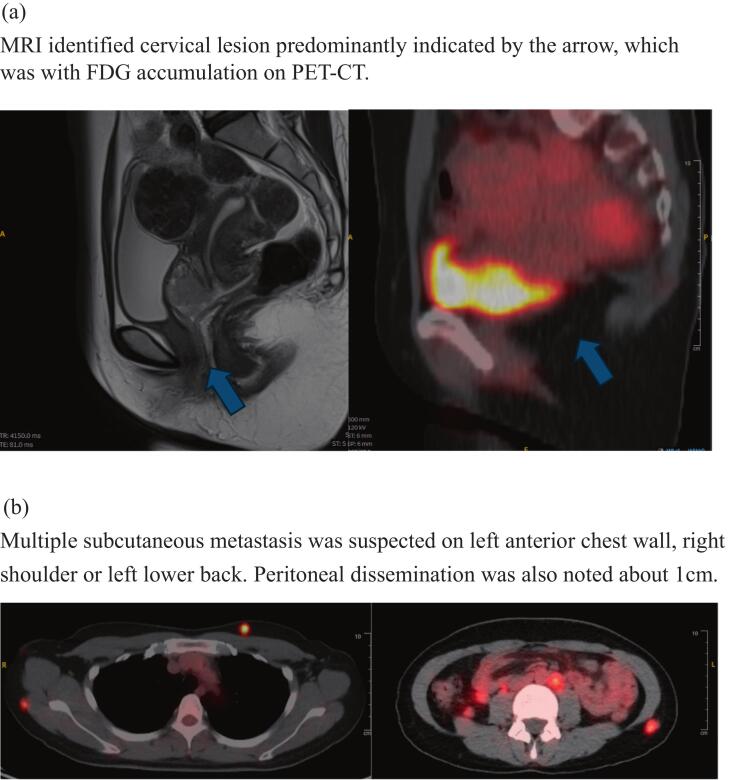
Pretreatment imaging. (a) T2-weighted sagittal MRI identified a cervical lesion (blue arrow), with FDG-avidity seen on PET-CT. (b) PET-CT revealed multiple subcutaneous metastases on the left anterior chest wall, right shoulder and left lower back. Multiple lesions were also visualized throughout the peritoneum. (For interpretation of the references to colour in this figure legend, the reader is referred to the web version of this article.)

Histopathological analysis showed atypical cells with a high nuclear-to-cytoplasmic (N/C) ratio arranged in nested or sheet-like patterns. Immunohistochemical analysis revealed positivity for synaptophysin and CD56 (Fig. 2), supporting a diagnosis of small cell neuroendocrine carcinoma (SCNEC). The combined positive score (CPS) was ≥1. Serum tumor markers were elevated (NSE: 47.0 ng/mL; ProGRP: 113.0 pg/mL). Based on these findings, the patient was diagnosed with stage IVB cervical SCNEC.

**Fig. 2:**
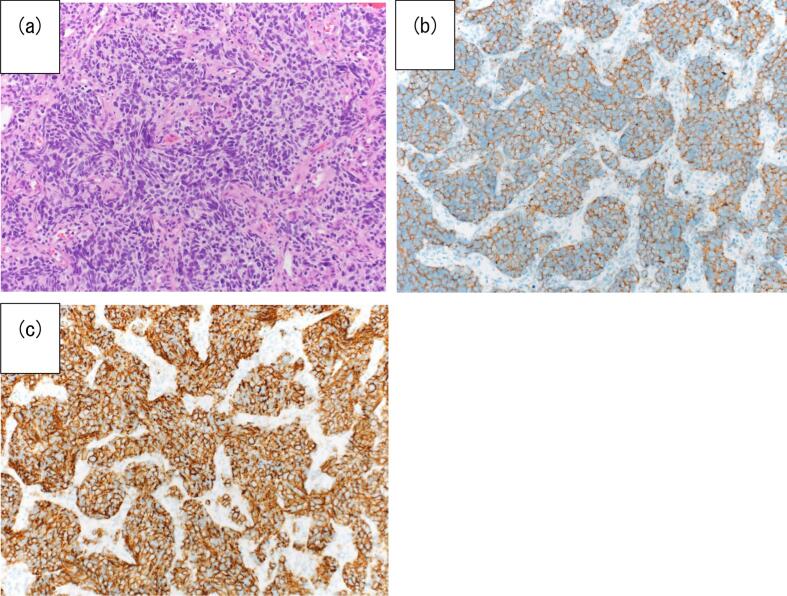
Histological findings of the cervical tumor (a) Photomicrograph showing atypical cells with a high N/C ratio (H&E stain, 20× objective lens). (b) Immunohistochemical staining for Synaptophysin (20× objective lens). (c) Immunohistochemical staining for CD56 (20× objective lens).

Systemic chemotherapy consisting of paclitaxel and carboplatin combined with bevacizumab and pembrolizumab was initiated (paclitaxel 175 mg/m^2^, carboplatin AUC 5, bevacizumab 15 mg/kg and pembrolizumab 200 mg intravenously every three weeks). After three cycles of treatment, the patient developed mild eczematous dermatitis of the fingers consistent with the Common Terminology Criteria for Adverse Events (CTCAE) Grade 2. The management for the dermatitis included oral olopatadine hydrochloride and topical betamethasone valerate. No grade ≥3 adverse events were observed during the seven total cycles of therapy.

After administration of 6 courses of chemotherapy, PET-CT scan was scheduled to assess residual tumor. Because of interval until its examination, one additional course was given, resulting in total seven courses of chemotherapy. Approximately 5 months after initial treatment, PET-CT showed complete resolution of the previously noted primary and metastatic FDG-avid lesions (Fig. 3a, b) and serum NSE and ProGRP levels normalized. Pelvic examination confirmed resolution of the cervical tumor, and cervical biopsy showed no evidence of residual malignancy, indicating complete remission.

**Fig. 3:**
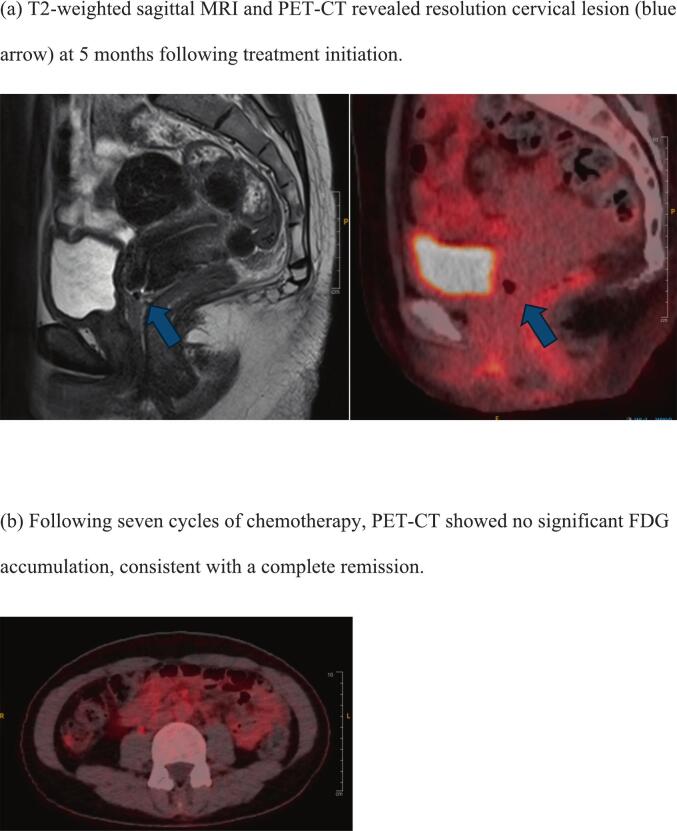
Post-treatment imaging (a) T2-weighted sagittal MRI and PET-CT revealed resolution cervical lesion (blue arrow) at 5 months following treatment initiation. (b) Following seven cycles of chemotherapy, PET-CT showed no significant FDG accumulation, consistent with a complete remission. (For interpretation of the references to colour in this figure legend, the reader is referred to the web version of this article.)

The patient has continued on maintenance therapy with bevacizumab and pembrolizumab (bevacizumab 15 mg/kg and pembrolizumab 200 mg both intravenously every three weeks) for 9 months, with continued disease control. Mild diarrhea (Grade 1 to 2) was observed and successfully managed with probiotics and Kampo medicine, keishikashakuyakuto (EKT-60).

## 3 Discussion

Cervical cancer is one of the most common gynecologic malignancies in Japan. According to Global Cancer Observatory (GLOBOCAN), cervical cancer remains a major global health burden, with approximately 662,000 new cases and 348,000 deaths reported worldwide in 2022. (International Agency for Research on Cancer, 2022).

Small cell neuroendocrine carcinoma (SCNEC) of the cervix is quite rare, accounting for only 0.5%–1.5% of invasive cervical neoplasms. It is characterized by rapid hematogenous or lymphatic metastasis and a poor prognosis (Wistuba et al., 1999, Miyoshi et al., 2023). According to previous report, most cases of cervical SCNEC are associated with human papillomavirus (HPV) infection, predominantly HPV18, with approximately 85% of cases HPV-positive across 403 reported cases in 32 studies (Castle et al., 2018). A systematic review and *meta*-analysis evaluating over 400 cases of SCNEC reported that 78% were positive for HPV16 or HPV18, with roughly half of these cases showing isolated HPV18 positivity (Castle et al., 2018). Our case exhibited positive p16 staining, suggesting an HPV-associated origin. Immunohistochemically, the tumor cells showed a dot–like staining pattern for CK AE1/AE3 and were positive for synaptophysin and CD56. These findings are consistent with a diagnosis of small cell neuroendocrine carcinoma (McCluggage et al., 2010). As to the tumor markers, NSE, neuron-specific enolase is well known as serum biomarker in neuroendocrine tumors. ProGRP, progasrin-releasing peptide is reported as a more sensitive and specific biomarker than NSE, associating with progression free survival and overall survival in patients with limited-stage small cell lung cancer (SCLC) receiving chemoradiotherapy (Gronberg et al., 2025).

Treatment options for cervical cancer include surgery, concurrent chemoradiotherapy or brachytherapy, systemic chemotherapy. Classically, platinum-based chemotherapy is the foundation of first-line chemotherapy treatment such as cisplatin or carboplatin combination with paclitaxel, and bevacizumab in advanced or recurrent cervical cancer. Although irinotecan and etoposide can be considered as second- or third-line treatments, their response rates are typically low. Recent advances include immune checkpoint inhibitors (ICIs), such as pembrolizumab or cemiplimab, or antibody–drug conjugate such as tisotumab vedotin, which have been approved for use in recurrent or metastatic cervical cancer (Monk et al., 2023, Vergote et al., 2024).

Given its rarity, a standardized treatment regimen for cervical SCNEC has not yet been established. For the prospect of common histotype, the treatment approaches have been extrapolated from the SCLC. Etoposide/cisplatin is the most widely used adjuvant chemotherapy regimen after surgery for cervical SCNEC (Miyoshi et al., 2023). Limited evidence exists for other chemotherapy regimens such as etoposide/platinum, paclitaxel/carboplatin, cisplatin/irinotecan, and cisplatin/ifosfamide/etoposide (Wistuba et al., 1999). A previous case report reported a complete response in a patient with recurrent cervical SCNEC treated with eight cycles of paclitaxel, carboplatin, and bevacizumab, after initial hysterectomy and adjuvant etoposide/cisplatin, with a 2-year recurrence-free interval (Nakao et al., 2020). Our case is, to the best of our knowledge, the first to report a complete remission in advanced cervical SCNEC using a combination of first-line chemotherapy (paclitaxel/carboplatin) with bevacizumab and pembrolizumab. This regimen may present a new treatment option for patients with advanced cervical SCNEC.

Bevacizumab, an anti-vascular endothelial growth factor (VEGF) antibody, serves a dual role beyond inhibition of angiogenesis. It promotes the normalization of the dysfunctional tumor vasculature, which in turn reduces intratumoral hypoxia and interstitial fluid pressure, thereby facilitating the effective infiltration of cytotoxic T lymphocytes into the tumor microenvironment. In addition, VEGF contributes to local immunosuppression by promoting the recruitment of regulatory T cells and myeloid-derived suppressor cells while inhibiting dendritic cell maturation (Ju et al., 2018). By counteracting these effects, bevacizumab may help restore a more “immune-favorable” environment. The synergistic effect of combining pembrolizumab with bevacizumab is likely mediated through the strategic modulation of the tumor microenvironment.

The KEYNOTE-826 trial showed improved overall survival (OS) of 9.6 months and progression-free survival (PFS) of 2.2 months for patients treated with pembrolizumab 200 mg plus platinum-based chemotherapy compared with placebo plus chemotherapy once every three weeks (Monk et al., 2023). The eligibility criteria comprised squamous cell carcinoma, adenosquamous carcinoma and adenocarcinoma in this trial, whereas SCNEC was not included. The CPS is considered to reflect the overall PD-L1 expression in the tumor microenvironment. Both a positive CPS and PD-L1 positivity are considered positive predictive factors for response to pembrolizumab (Monk et al., 2023). In cervical SCNEC, PD-L1 expression is reported in 25% of 16 cases with a CPS ≥1 (Yamada et al., 2025). Past phase 2 basket trial showed only limited activity of pembrolizumab alone treatment for the recurrent SCNEC of the lower genital tract, 6 cervical and 1 vulvar cancer. (Frumovitz et al., 2020) 14.3% (1/7) responded stable disease and the remaining cases resulted in progression disease. In our case, CPS was ≥1, suggesting that it was likely to respond favorably to pembrolizumab.

In other primary organprimary organ SCNEC, such as pulmonary SCNEC, PD-L1 expression, although generally low, has been reported to correlate with meaningful responses to ICI agents (Paz-Ares et al., 2019). For extensive-stage SCLC, standard therapy as a first-line is ICI and antiangiogenetic agent combined with etoposide-platinum chemotherapy (Wang et al., 2025). In SCLC, VEGF is overexpressed and is associated with poor prognosis and plays a fundamental role in tumor growth and spread. Then positive synergic effects in chemoimmunotherapy are transforming the treatment of SCLC. According to this *meta*-analysis, best OS and PFS were achieved with benmelstobart (a PD-L1 antibody) plus anlotinib (an antiangiogenic agent) and etoposide/carboplatin. SCLC and cervical SCNEC share important epidemiologic and biological features, including their highly aggressive behavior, inactivation of TP53 and RB1, and expression of ASCL1 and other master regulators of neuroendocrine differentiation. (Baine et al., 2020, Miyoshi et al., 2023) Therefore, it is reasonable to assume that they have similar treatment responsiveness. In our case, the tumor exhibited a CPS ≥1, and bevacizumab-based chemotherapy may have further modulated the tumor microenvironment to enhance immune infiltration as mentioned above. The combination of anti-angiogenic and immune checkpoint inhibition therapies may have therefore been critical in eliciting the effective antitumor immune response. These findings suggest that PD-L1/CPS expression and tumor microenvironmental features may help identify SCNEC patients who could benefit from ICI-containing regimens.

In recent molecular study, transcription factors such as ASCL1 and signaling pathways including YAP1 have been shown to define distinct molecular subtypes of SCNEC, each with potentially different therapeutic vulnerabilities (Baine et al., 2020). These insights have informed ongoing clinical trials targeting these pathways, highlighting the growing potential for biomarker-driven treatment strategies in SCNEC.

The chemotherapy regimen used in this patient, which included an ICI, may represent a promising strategy to improve outcomes for cervical SCNEC. Further prospective studies are warranted to identify the most effective treatment approaches for this rare and aggressive tumor type.

## 4 Conclusion

To our knowledge, this is the first case report of advanced cervical SCNEC demonstrating a complete response to combination paclitaxel and carboplatin chemotherapy with pembrolizumab and bevacizumab. This combination regimen could be a novel and effective therapeutic option for the treatment for cervical SCNEC.

## 5 Declaration of generative AI and AI-assisted technologies in the manuscript preparation process

During the preparation of this work, the author used chat GPT to improve the clarity and readability of the English language. The author reviewed and edited the content as needed and takes full responsibility for the content of the published article.

Written informed consent was obtained from the patient for publication of this case report and accompanying images.

## 6 Ethics approval and consent to participate and consent for publication

Written informed consent was obtained from the patient, and this case report was approved by the Institutional Review Board of Osaka International Cancer Institute according to the ethical standards laid down in the Declaration of Helsinki.

## CRediT authorship contribution statement

**Reisa Kakubari:** Writing – original draft, Investigation, Data curation, Conceptualization. **Yui Nonokawa:** Investigation, Data curation. **Yasuyuki Morita:** Resources, Investigation. **Masayo Hosokawa:** Resources, Investigation. **Keisuke Ogimoto:** Visualization, Investigation. **Eri Yamabe:** Visualization, Resources. **Miho Kitai:** Writing – review & editing, Supervision, Conceptualization.

## Declaration of competing interest

The authors declare that they have no known competing financial interests or personal relationships that could have appeared to influence the work reported in this paper.
